# A Low-Cost, High-Throughput Digital Image Analysis of Stain Patterns on Smoked Cigarette Filter Butts to Estimate Mainstream Smoke Exposure

**DOI:** 10.3390/ijerph181910546

**Published:** 2021-10-08

**Authors:** Clifford H. Watson, Jane Yan, Stephen Stanfill, Liza Valentin-Blasini, Roberto Bravo Cardenas, Benjamin C. Blount

**Affiliations:** U.S. Division of Laboratory Sciences, Centers for Disease Control and Prevention, 4770 Buford Highway, NE MS F-55, Atlanta, GA 30341, USA; xay3@cdc.gov (J.Y.); zsa1@cdc.gov (S.S.); lbv5@cdc.gov (L.V.-B.); zlg9@cdc.gov (R.B.C.); bkb3@cdc.gov (B.C.B.)

**Keywords:** cigarette, filter butts, nicotine, tar, smoke exposure

## Abstract

Standard machine smoking protocols provide useful information for examining the impact of design parameters, such as filter ventilation, on mainstream smoke delivery. Unfortunately, their results do not accurately reflect human smoke exposure. Clinical research and topography devices in human studies yield insights into how products are used, but a clinical setting or smoking a cigarette attached to such a device may alter smoking behavior. To better understand smokers’ use of filtered cigarette products in a more natural environment, we developed a low-cost, high-throughput approach to estimate mainstream cigarette smoke exposure on a per-cigarette basis. This approach uses an inexpensive flatbed scanner to scan smoked cigarette filter butts and custom software to analyze tar-staining patterns. Total luminosity, or optical staining density, of the scanned images provides quantitative information proportional to mainstream smoke-constituent deliveries on a cigarette-by-cigarette basis. Duplicate sample analysis using this new approach and our laboratory’s gold-standard liquid chromatography/tandem mass spectrometry (LC/MS/MS) solanesol method yielded comparable results (+7% bias) from the analysis of 20 commercial cigarettes brands (menthol and nonmentholated). The brands varied in design parameters such as length, filter ventilation, and diameter. Plots correlating the luminosity to mainstream smoked-nicotine deliveries on a per-cigarette basis for these cigarette brands were linear (average R^2^ > 0.91 for nicotine and R^2^ > 0.83 for the tobacco-specific nitrosamine NNK), on a per-brand basis, with linearity ranging from 0.15 to 3.00 mg nicotine/cigarette. Analysis of spent cigarette filters allows exposures to be characterized on a per-cigarette basis or a “daily dose” via summing across results from all filter butts collected over a 24 h period. This scanner method has a 100-fold lower initial capital cost for equipment than the LC/MS/MS solanesol method and provides high-throughput results (~200 samples per day). Thus, this new method is useful for characterizing exposure related to filtered tobacco-product use.

## 1. Introduction

Cigarette smoking remains a leading preventable cause of premature death in the United States [1]. Tobacco smoke contains a complex mixture of more than 4000 chemicals [2]. Inhaling harmful chemicals from cigarette smoke is associated with increased risk of cancer, birth defects, and heart disease [2,3,4]. The International Agency for Research on Cancer [5] groups individual chemicals and chemical mixtures according to their carcinogenicity in humans, and has classified whole cigarette smoke as a known human carcinogen related to numerous cancers. Understanding specific chemical deliveries from mainstream cigarette smoke under natural conditions is important to help assess nicotine addiction and health risks related to other chemical exposures from cigarette use. 

Determining the mainstream cigarette smoke delivery of nicotine and other harmful chemicals under real-world conditions is challenging. Modern smoking machines that use fixed smoking regimens are often used to establish regulatory limits for specific cigarette yields. Such measurements can be useful to make product-to-product comparisons, but are poor predictors of actual smoker exposure because human smoking behaviors are typically much more diverse [6,7,8]. Machine smoking regimens use fixed puff volumes, durations, and intervals. Smokers, however, do not smoke each cigarette the same way. Different smokers, using the same brand, can have very different smoking behaviors based on their individual, time-specific nicotine cravings [9]. By changing puff volume, time interval between puffs, and the potential filter vent-hole obstruction with lips or fingers, smokers can easily influence their inhalation of smoke constituents [10,11,12]. 

Previous researchers have used many approaches for examining exposures to nicotine and other harmful or potentially harmful constituents in mainstream tobacco smoke. These methods have included questionnaires [13], the use of topography devices to record air flow drawn through a cigarette during a smoking session [14], analysis of urine or saliva for exposure biomarkers [15], and measurement of exhaled carbon monoxide [11]. While these methods provide useful data, nonbiomarker methods can vary in accuracy and interpretation [16,17,18] and alter how a person smoke [19]. Furthermore, collecting physiological fluids for biomarker measurement can be invasive and biomarker concentrations are time-averaged measures (not specific to each cigarette smoked) influenced by time of last use and individual metabolic differences [16,19]. 

Solanesol (and other mainstream smoke constituents) accumulates in cigarette filter butts during smoking and can be measured to accurately assess the intensity with which each cigarette is smoked [20,21,22]. Such analyses can provide crucial data on nicotine and smoke toxicant deliveries to better understand addiction and other harms related to smoking. Additionally, using smoked filter butts to characterize how people smoke cigarettes and examine the body burden of harmful chemicals can help determine whether new regulations meaningfully reduce or otherwise impact exposure. Multiple research groups have estimated smoke intake by using various forms of filter analyses [23,24,25,26,27,28,29,30]. 

Filter-butt analysis approaches are also useful because data can be obtained on a per-cigarette basis under more natural, real-world conditions. Such information provides data related to smoke constituent deliveries as well as behavioral use patterns. Our laboratory successfully used this method to examine the difference in deliveries of nicotine and tobacco-specific nitrosamines [31], the influence of menthol on smoking behavior [32], differences in smoking behaviors in a clinical setting [33], and changes in smoking when cigarette nicotine content is reduced [34,35]. This new luminosity-based, digital-image approach is a low-cost, high-throughput method that provides comparable results to our established liquid chromatography/tandem mass spectrometry (LC/MS/MS) solanesol method [21,22].

## 2. Materials and Methods

### 2.1. Cigarette Styles Analyzed

To demonstrate the feasibility and utility of this approach, we purchased 20 cigarette brands from a local vendor (Lab Depot, Dawsonville, GA, USA). The cigarettes were selected with a range of different physical properties to reflect the diversity of products on the market. These cigarette brands were of two different lengths: ten king-size brands and ten 100s brands (~84 mm and ~100 mm, respectively). The cigarettes were a mixture of mentholated and nonmentholated brands (11 and 9 brands, respectively) with filter-ventilation levels ranging from 0% to 56%. Eighteen brands had the standard cigarette diameter and two brands were slim varieties (7.5 mm and 5 mm, respectively). Upon receipt of the products from the vendor, unopened cigarette packs were logged into the products database, labeled, placed in resealable storage bags, and stored at −70 °C until analysis. 

### 2.2. Correlating Filter-Stain Patterns with Mainstream Smoke Deliveries

Cigarettes were conditioned in an environmental chamber at 22 °C and 60% humidity for a minimum of 24 h prior to smoking. An SM450 16-port linear smoking machine (Cerulean, Milton Keynes, UK) was used for machine smoking. Systematic variation of puff volume, puff frequency, and filter-ventilation blocking provided a wide range of smoking conditions and resultant mainstream smoke deliveries. Cigarettes were smoked under an ISO standard smoking regimen (5 cigarettes per pad, 35 mL puff, 2s puff duration, 60s puff interval, filter vent holes unobstructed) and an intensive regimen (3 cigarettes per pad, 55 mL puff, 2s puff duration, 30s puff interval, filter vent holes completely blocked). All measurements were made in triplicate. For ISO smoking, total particulate matter (TPM) was collected from smoking runs with 2 puffs, 4 puffs, and normal butt termination (puff count when cigarette coal reached a 3 mm distance from the filter paper overwrap). For intense smoking, TPM was collected from smoking runs with 4 puffs, 6 puffs, and normal butt termination (puff count when cigarette coal reached a 3 mm distance from the filter paper overwrap). This smoking protocol provided a roughly uniform distribution of six variable mainstream smoke deliveries for each cigarette brand.

**Figure 1:**
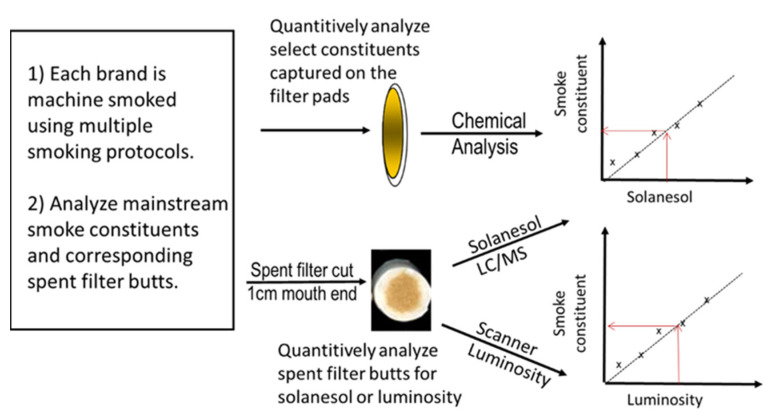
Protocol for measuring human smoke exposure using digital imaging and chemical analysis of spent cigarette filters.

The mainstream smoke TPM for each smoking regimen/puff count combination was measured for nicotine [36] and the tobacco-specific nitrosamine 4-(methylnitrosamino)-1-(3-pyridyl)-1-butanone (NNK) [37]. The corresponding cigarette butts were collected for subsequent digital and solanesol analyses (Figure 1). A scalpel was used to cut the butt at a length of 1 cm from the mouth end, preserving ventilation holes so their effects could also be captured. The cleanly cut inner surface of the filter butt was digitally scanned at 600 dpi using a standard commercial CCD-based, color flatbed scanner (HP ScanJet 4670, HP Inc., Palo Alto, CA, USA; https://support.hp.com/us-en/product/hp-scanjet-4670-scanner-series/303640/manuals, accessed on 31 August 2021). The scanner’s OEM software was used without modification to scan the images; autocorrection exposure and contrast features were all turned off. All images were collected using 600 dpi scanning resolution. To provide consistent images and enable accurate scan-to-scan comparisons, a reference target strip, Kodak Q-13, Eastman Kodak Company, Rochester, NY, USA; https://www.kodak.com/en/motion/page/color-separation-guides-and-gray-scales, accessed on 31 August 2021) was included with each scan to reproducibly set the white and black points for each scan. After scanning, the butts were analyzed by our quantitative LC/MS/MS solanesol method [21] to allow direct comparison of the two approaches.

**Figure 2:**
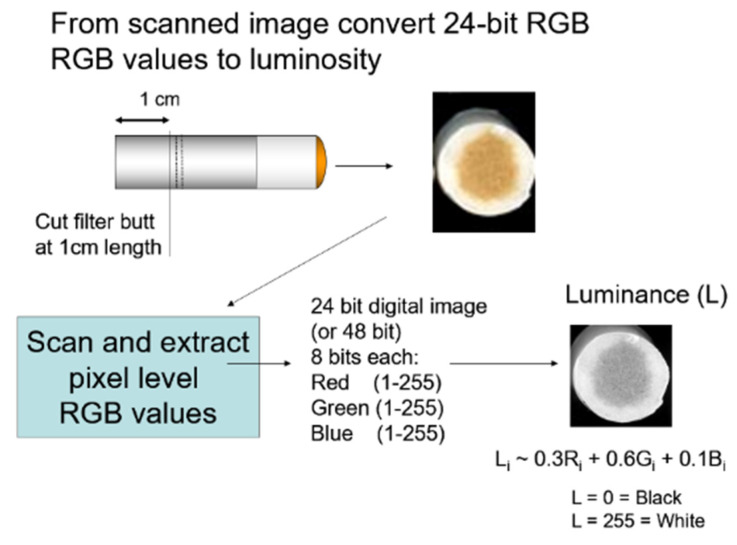
Example of captured image and determination of the corresponding luminosity value (1-255).

Scanning the inside surface of a 1 cm, cleanly cut filter butt provides a clean image free of debris (e.g., tobacco flakes, dust, and lipstick). The cut position is limited because the filter ventilation holes in many cigarette brands are ~12–13 mm from the mouth end. We experimented with shorter cuts (data not shown) but found the 10 mm cut optimal as it provided the maximum sample to extract and analyze in order to optimize sensitivity. Each individually scanned filter-butt image is analyzed using software developed using Visual Basic (code available upon request) to determine the corresponding luminance (Figure 2). The luminance is then correlated to the smoke delivery from each butt. Because of the differences in filter designs (e.g., length, fiber denier, porosity, and ventilation), the filter efficiency varies from brand to brand. Brand-specific correlation curves were thus constructed relating the luminosity of each filter’s image to the corresponding measured mainstream smoke deliveries.

The luminosity was calculated from the 24-bit RGB representation values (8-bits each for red (R), green (G), and blue (B) measured by the scanner’s software and using the following equation:

Luminosity = 255 − (0.3 × R + 0.6 × G + 0.1 × B)(1)

The normal value for luminosity (0.3 × R + 0.6 × G + 0.1 × B) was subtracted from 255 (2^8^) so that darker values (higher deliveries of mainstream smoke) would correspond to larger values. Thus, for the white cellulose acetate filters the luminosity was near zero for unsmoked butts. As more tar collects in the filter, the staining pattern darkens. The luminosity-derived value could increase from zero to a maximum value of 255 (corresponding to a ~3 mg nicotine/cigarette upper limit of measurable delivery). The relationship between measured filter-butt luminosity and the range of mainstream smoke nicotine deliveries for the 20 filtered cigarette brands included can be approximated using a linear regression model.

## 3. Results

Correlation curves were obtained for the 20 cigarette brands by measuring the mainstream smoke deliveries for nicotine and the tobacco-specific nitrosamine, NNK under a range of smoking conditions (see Experimental Section and Figure 1). The spent cigarette filter butts used to generate the mainstream smoke samples were analyzed for the corresponding scanner-based luminosity and solanesol content. Because scanning the filter butts for capturing luminosity values is nondestructive, the same butts were used for the solanesol measurements. Correlation curves linking the filter measurements to corresponding mainstream smoke deliveries were obtained using the same procedure as our previous solanesol analyses [21]. In all cases, we used a simple linear regression to evaluate linearity based on a coefficient of determination (R^2^) and the regression model equation for each cigarette brand (summarized in Table 1).

**Figure 3:**
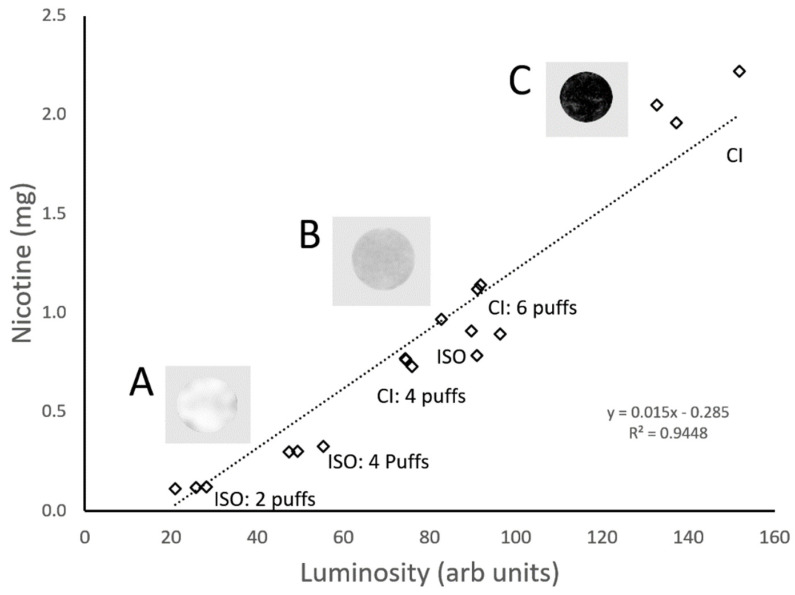
Correlation curve relating the measured mainstream smoke deliveries from a low-delivery cigarette brand and corresponding luminosity levels from the filter butts: Inserts A, B, and C represent staining patterns from filters obtained using two ISO puffs, two CI puffs, and full CI smoking conditions, respectively.

The resulting correlation curves linking the mainstream smoke deliveries and luminosity values were determined using linear regressions over all the smoking protocols used. An example correlation plot (Figure 3) demonstrates the relationship between the measured mainstream smoke nicotine deliveries for a typical cigarette brand using a range of smoking conditions with the corresponding luminosities measured from the respective cigarette filter butts. The correlation plots linked the measured mainstream nicotine values and the luminosities measured from the stain patterns. For this example, a good linear fit of the regression line had an R^2^ of 0.94. An average R^2^ value of 0.91 was obtained for nicotine correlation plots from the 20 cigarette brands. The linear regression fits for luminosity correlation curves were comparable to those achieved with the LC/MS/MS solanesol method (Table 1). Cigarette design differences such as filter length, paper porosity, and tobacco mass necessitated the generation of brand-specific correlation curves as is the case for the solanesol method. 

**Figure 4:**
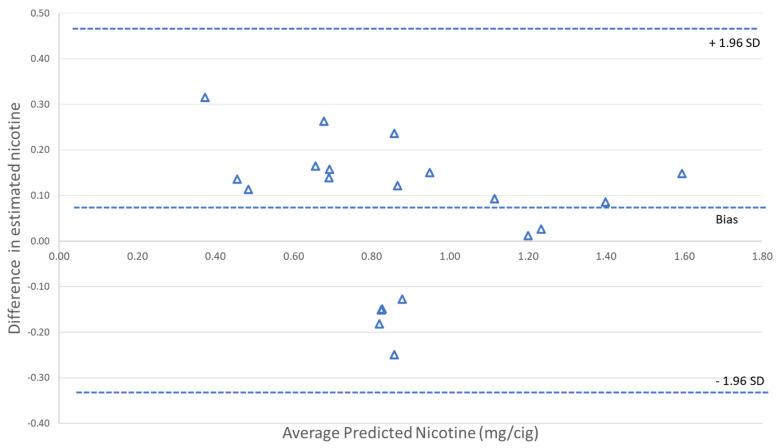
Bland-Altman plots showing agreement between the standard solanesol analytical methodology and the scanner-based luminosity approach for predicting ISO nicotine deliveries for 20 cigarette brands. Δ = Difference in estimated nicotine (X_solanesol_ – X_scanner_).

The corresponding slopes and y-intercepts were used to determine the estimated ISO nicotine deliveries from butts using the standard linear-regression equations for both the solanesol and luminosity approaches. A Bland–Altman plot [38] was then generated to compare the estimated ISO nicotine deliveries for the 20 cigarettes brands using each brand’s specific correlation curve (Figure 4). The mainstream ISO machine-smoked nicotine deliveries showed good agreement between the two methods. The Bland–Altman plots also indicated that the scanner method had a +7% bias relative to the solanesol method; the bias was mostly observed in the lower nicotine-delivery range. We analyzed the data using a multivariate analysis to see which, if any, physical properties of the cigarettes (tobacco weight, cigarette length, filter length, diameter, and filter ventilation) contributed to the bias. A weak but non-statistically significant relation (*p* > 0.05) was observed between the bias and filter length. This could be related to the lower delivery cigarettes with higher filter ventilation that have very low yields under the ISO smoking regime at low puff numbers. We attribute this bias to the much higher dynamic range of the MS relative to the inexpensive flatbed scanner. The LC/MS/MS solanesol method has a much wider dynamic range (~10^6^) compared to the 8-bit luminosity-based approach (~10^2^).

A potential limitation was previously published questioning whether the color stain patterns of discarded cigarettes could change or fade over time [39]. We investigated this issue for our current approach using a time-dependent stability analysis. A series of commercial cigarette butts were machine smoked, collected in bulk, and stored at room temperature and a −20 °C freezer in a resealable storage bag. The butts were analyzed using the scanner-based method over the course of four weeks. Using the Student’s *t*-test to compare measurements over time, we observed no significant statistical change in measured luminosity over time or storage conditions.

The scanner approach was successfully piloted and compared with other measures in an analysis of reduced-nicotine-content cigarettes to help estimate the potential for compensatory smoking among intermittent smokers when using these cigarettes [40]. The data presented here indicate that the scanner approach is applicable to a wide range of commercial cigarettes covering a range of nicotine deliveries, cigarette filler amounts, and filter lengths. To demonstrate the utility with other mainstream smoke constituents, we also measured the corresponding luminosity and LC/MS/MS solanesol correlations to the mainstream smoke deliveries of NNK (Table 1). The R^2^ values for NNK were not as precise as those for nicotine, likely because the concentration of NNK relative to nicotine is about 1000-fold lower. 

In general, we found good agreement in linearity of the correlation curves across the 20 brands examined for the estimated nicotine and NNK mouth-level intake between the new luminosity measurement and our established solanesol methodology. 

The scanner luminosity method and the MS/LS/LS solanesol method each has advantages and disadvantages. As noted above, the solanesol approach has a much higher dynamic range. On the other hand, the initial infrastructure costs for the luminosity measurements are much lower compared to the solanesol method. For the luminosity method, the main requirements are a scanner, reference target, desktop computer, and software to analyze the stain patterns (2021 equipment cost estimate is ~USD 3000). The solanesol method uses an LC/MS/MS instrument and automated sample preparation station with an estimated equipment cost of ~USD 400,000 (2021). Additionally, the solanesol method requires a highly skilled operator, so personnel costs would be higher than those needed for the simpler scanner method. However, some measurement costs are shared for both approaches. For example, as noted above, each correlation curve is brand specific, requiring 18 smoke runs (6 conditions: three each ISO and three each intense, performed in triplicate). Generating these correlation curves can incur significant costs depending on the number of brands analyzed. 

A benefit of our filter-butt approaches luminosity, mass spectrometry [21,22] or UV absorbance [30] is the opportunity to probe nicotine delivery or other select constituent deliveries under natural conditions. This can be done on a per-cigarette basis or a daily-dose basis by summing results from all butts collected over a 24 h period. We have successfully used our solanesol-based approach to examine difference in deliveries and smoking behavior as a function of menthol [32], tobacco blends [29,31], and the use of reduced-nicotine-content cigarettes [34,35] for a variety of mainstream smoke constituents. However, the scanner-based method could produce comparable data while saving time and money. 

One potential application of the luminosity method is as a screening approach to examine relative changes in smoking patterns within specific brands. For example, this approach could be useful for determining if relative changes in smoking intensity are related to specific cravings, situations, locations, reactions to various stimuli, concurrent use of other nicotine products, or various cessation interventions. As noted by Jarvis et al. (2001) humans do consume cigarettes as predicted by standardized smoking machine measurements [6]. The correlation curves linking standard smoking machine yields to those achieved under naturalistic smoking conditions provides improved delivery estimates. However, the filter-butt analysis does not reflect the degree of inhalation. A commonly used means to examine changes in smoking is to look at the number of cigarettes smoked per day. The exposure “gold-standard”, biomarkers of exposure, may not decrease proportionally as the number of cigarettes smoked per day decreases [41]. A likely explanation is that smokers compensate for fewer cigarettes by smoking those cigarettes more intensely to achieve a desired nicotine dose. 

In many cases, the low-cost luminosity approach could be useful to probe behavior-related changes in smoking intensity or to test various hypotheses before incurring the expense of making appropriate biomarker measurements. Biomarker measurement has the advantage of capturing an internal measure of exposure that is not biased by subjective reporting or forgetting to save/return used cigarette filter butts for analysis. However, in cases where biomarker analysis is cost-prohibitive due to sample size or budgetary constraints, the filter analysis approach could provide much-needed information. Furthermore, biomarker data also are time-averaged based on the half-life of the biomarker; thus, biomarker data lack the time-dependent resolution of the filter methods for understanding exposure on a per-cigarette basis. Analysis of filters is not time-dependent on last cigarette use. Additionally, filter analysis can provide both intake per cigarette and daily delivery information by summing across cigarette butts to determine total intake over a 24 h cycle. The filter-based approaches (e.g., via measuring luminosity, solanesol, UV absorbance, or other approaches) have value for probing natural, real-world smoking deliveries and behaviors. These include, but are not limited to, providing information on a smoker’s responses to various stimuli, examining effectiveness of treatment options, probing compensation behavior, studying changes in smoking patterns with modified cigarettes, and examining smokers’ reactions to regulatory changes in product design.

## 4. Conclusions

The digital-image, scanner-based approach for determining the luminosity of staining patterns in cigarette filter butts provides comparable data to our established solanesol method for estimating exposures to select mainstream smoke constituents. We found good agreement with comparable linear responses among the brand-specific constituent correlation curves between the luminosity and solanesol methods for filtered cigarettes. Biomarker measurements will always be the gold standard for exposure assessment. However, used cigarette-filter approaches such as the luminosity method presented here can generate supplemental information in terms of delivery per cigarette and provide an opportunity to probe changes in smoking behavior under various conditions.

## Figures and Tables

**Table 1:** Comparison of luminosity and solanesol average correlation curve fits for nicotine and NNK for 20 brands of commercial cigarettes.

	Nicotine		NNK ^1^	
	Luminosity	Solanesol	Luminosity	Solanesol
R^2^	0.905	0.893	0.831	0.842
Stdev	0.050	0.079	0.169	0.100
RSD (%)	5.6	8.9	20.4	11.8

^1^ NNK: 4-(methylnitrosamino)-1-(3-pyridyl)-1-butanone.
